# Supplementary material for: Clear variation of spin splitting by changing electron distribution at non-magnetic metal/Bi2O3 interfaces
Source: Sci Rep. 2018 Apr 3;8:5564. doi: 10.1038/s41598-018-23787-4 (PMC5883063; doi:10.1038/s41598-018-23787-4)
Supplement: Supplementary file 1 — Supplemental information [file 41598_2018_23787_MOESM1_ESM.pdf]

Supplemental information

Clear variation of spin splitting by changing electron distribution at non-magnetic metal/Bi_2O_3 interfaces

H. Tsai[1], S. Karube[1], K. Kondou[2*], N. Yamaguchi[3], F. Ishii[4] and Y. Otani[1, 2,*)]

[1] *Institute for Solid State Physics, University of Tokyo, Kashiwa 277-8581, Japan*

[2] *Center for Emergent Matter Science, RIKEN, 2-1 Hirosawa, Wako 351-0198, Japan*

[3] *Division of Mathematical and Physical Sciences, Graduate School of Natural Science and Technology, Kanazawa University, Kanazawa 920-1192 Japan*

[4] *Faculty of Mathematics and Physics, Institute of Science and Engineering, Kanazawa University, Kanazawa 920-1192, Japan.*

*Correspondence authors: kkondou@riken.jp, yotani@issp.u-tokyo.ac.jp

Table of contents

1. **Influence of spin Hall effect in bulk**
2. **Film crystallinity**
3. **Frequency dependence of spin-to-charge conversion efficiency and Rashba parameter**
4. **First-principles calculation results and spin textures**

1. Influence of spin Hall effect in bulk

When measuring the spin-to-charge (S-C) conversion at the Bi_2O_3 interface of Ag, Cu, and Al, the spin Hall effect of these NM materials is negligible small. However, in Au/Bi_2O_3 case spin Hall angle of Au is one order larger than others and makes notable contribution. For analyzing Py/Au/Bi_2O_3 sample, the contribution of SHE of Au and IEE in Au/Bi_2O_3 interface need to be separated. Firstly, we measure the spin Hall angle of Au by measuring S-C conversion in Py/Au/Al_2O_3 sample. By solving the spin diffusion equation with the boundary condition that spin current is zero at Au/Al_2O_3 interface, the spin current flowing in the Au layer is

$$J_{\mathrm{s}}(y) = \frac{\sinh[(t_{\mathrm{N}} - y)/\lambda_{\mathrm{N}}]}{\sinh(t_{\mathrm{N}}/\lambda_{\mathrm{N}})} J_{\mathrm{s}}^{0} \quad \text{(S1)}$$

where t_{N}, and λ_{N} are the thickness of NM layer, and the spin-diffusion length of NM layer, respectively. J_s^0 is the spin current injected at Py/Au interface which is shown in eq. (3). Here, we use λ_{N} = 35 nm from a reported value [*Phys. Rev. B* **88**, 064414]. The average spin current density is $\langle J_{\mathrm{s}} \rangle = \frac{1}{t_{\mathrm{N}}} \int_0^{t_{\mathrm{N}}} J_{\mathrm{s}}(y)$ and the average charge current density in three dimension is $\langle J_{\mathrm{c}} \rangle = \theta_{\mathrm{SH}} \langle J_{\mathrm{s}} \rangle$. Therefore, the spin Hall angle θ_{SH} can be calculated by

$$\langle J_{\mathrm{c}} \rangle = \theta_{\mathrm{SH}} \left(\frac{2e}{\hbar}\right) \frac{\lambda_{\mathrm{N}}}{t_{\mathrm{N}}} \tanh\left(\frac{t_{\mathrm{N}}}{2\lambda_{\mathrm{N}}}\right) J_{\mathrm{s}}^{0} \quad \text{(S2)}$$

As the result, θ_{SH} of Au is +0.40±0.07%, which is in a good agreement with reported value measured by spin-pumping method . The next step is considering the interface effect of Au/Bi_2O_3. Because some spin current is injected into the Au/Bi_2O_3 interface, the backflow of spin current is reduced and the injected spin current increased at Py/Au interface, i.e. $J_{\mathrm{s(Au/Bi_2O_3)}} = J_{\mathrm{s(Au/Al_2O_3)}} + \Delta J_{\mathrm{s}}$ and $\Delta J_{\mathrm{s}} > 0$. Since the backflow of spin current decays from y = t_{N} to y = 0, that is ΔJ_s(y) = $\Delta J_s^0 e^{y/\lambda_{\mathrm{N}}}$ and ΔJ_s^0 = ${J_{\mathrm{s}}}^0{}_{(\mathrm{Au/Bi_2O_3})} - {J_{\mathrm{s}}}^0{}_{(\mathrm{Au/Al_2O_3})}$. The spin current in Au/Bi_2O_3 can be expressed as

$$J_{\mathrm{s}}(y) = \frac{\sinh[(t_{\mathrm{N}} - y)/\lambda_{\mathrm{N}}]}{\sinh(t_{\mathrm{N}}/\lambda_{\mathrm{N}})} J_{s\,(\mathrm{Au/Al_2O_3})}^{0} + \Delta J_s^0 e^{y/\lambda_{\mathrm{N}}} \quad \text{(S3)}$$

Again, the average spin current density is $\langle J_s \rangle = \frac{1}{t_{\mathrm{N}}} \int_0^{t_{\mathrm{N}}} J_s(y)$. We assumed that the θ_{SH} of the Au bulk in Au/Bi_2O_3 and Au/Al_2O_3 are approximately equal since the typical thickness of interface layer is only 0.4 nm. By separating the contribution of ISHE and IEE, the 3D charge current density $\langle J_c \rangle$ can be expressed as

$$\langle J_{\mathrm{c}} \rangle = \langle J_{\mathrm{s}} \rangle \theta_{\mathrm{SH}} + J_{\mathrm{s(interface)}} \times \lambda_{\mathrm{IEE}} t_{\mathrm{N}} \quad \text{(S4)}$$

and then the λ_{IEE} of Au/Bi_2O_3 interface is derived.

2. Film crystallinity

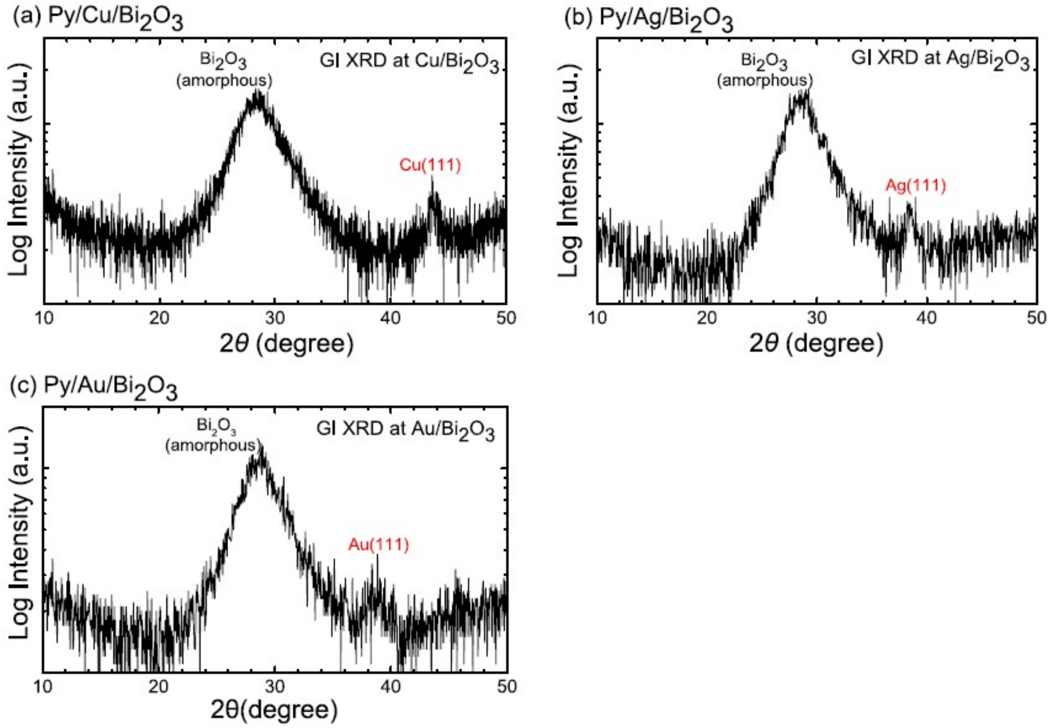

Figure S1| X-ray diffraction results

Grazing incident (GI) XRD spectrums at (a)Py/Cu/Bi_2O_3 (b)Py/Ag/Bi_2O_3 (c) Py/Au/Bi_2O_3 samples.

We use Grazing incident X-ray diffraction (GI XRD) to get the crystallinity information at NM/Bi_2O_3 interface of each Py/NM/Bi_2O_3 samples. For the NM layer (NM = Ag, Cu, and Au), at the interfaces Ag(111), Cu(111), and Au(111) structure are observed. These results suggest that the NM/Bi_2O_3 (NM = Ag, Cu, and Au) interfaces may have similar interface structure and therefore the strong NM dependence may not come from the crystal structure difference.

3. Frequency dependence of S-C conversion coefficient and effective Rashba parameter

We also investigated the frequency dependence of S-C conversion. Because the spin current generated by spin pumping in average is a dc spin current, the S-C conversion and Rashba parameter at NM/Bi_2O_3 should not depend on the frequency of rf field. As expected, by measuring the same Py/Cu/Bi_2O_3 sample at 6,7,8, and 9 GHz, λ_{IEE} is 0.19±0.005nm and $\alpha_{\mathrm{R}}^{eff}$ is 0.27±0.007 (eV·Å). The error is 2.6% which may come from the measurement and data fitting.

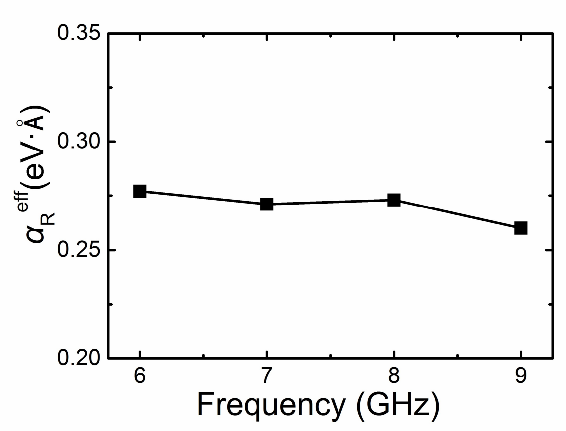

Figure S2|Frequency dependence of Rashba parameter

4. First-principles calculation results and spin textures

Fig. S3(a)-(c) shows the band structure for the NM(111)/$\boldsymbol{\alpha}$-Bi_2O_3 systems, where the symmetry points (Γ, C, X) are those in the first Brillouin zone shown in Fig. S4(a). There is a free-electron-like band around C-point near the Fermi energy for each system, and its Rashba spin splitting is anisotropic. A trend in the Rashba spin splitting is corresponding to experimental one, and we obtained the Rashba coefficients $\boldsymbol{\alpha}_{\mathbf{R}}$ as the average of the ones along CΓ and CX line around C-point. Our calculated $\boldsymbol{\alpha}_{\mathbf{R}}$ are 0.91, 0.50 and 0.29 for NM = Cu, Ag and Au, respectively, in units of eV·Å.

Fig. S4(a) shows the schematic of the first Brillouin zone of α-Bi_2O_3. Fig. S4(b)-(d) shows the spin textures for the NM(111)/α-Bi_2O_3 system. The anisotropic Rashba spin structures are shown for NM = Cu (Fig. S4 (b)) and for NM = Ag (Fig. S4 (c)), while the non-Rashba type spin structures are shown for NM = Au (Fig. S4 (d)). Since α-Bi_2O_3 is monoclinic ($P2_1/c$, No. 14) and C-point is Brillouin zone-boundary, each system has no 4-fold rotational symmetry (around C-point) that makes Fermi surface and spin textures isotropic. The anisotropic Rashba spin vortices for NM = Cu and Ag are opposite to each other (e.g. The inner (outer) vortex for NM = Cu is the clockwise (anti-clockwise), while that for NM = Ag is the anti-clockwise (clockwise).), which may support our experimental result that the sign of $\alpha_R{}^{eff}$ in Ag/Bi_2O_3 is positive while that in Cu/Bi_2O_3 is negative. For NM = Au, there are non-Rashba type spin splitting. This may be due to strong SOC of Au. On the other hand, in the experiment, a symmetric circular spin structure was observed by angle dependence results but not an anisotropic one, because the amorphous Bi_2O_3 results in a symmetric potential in x-y plane.

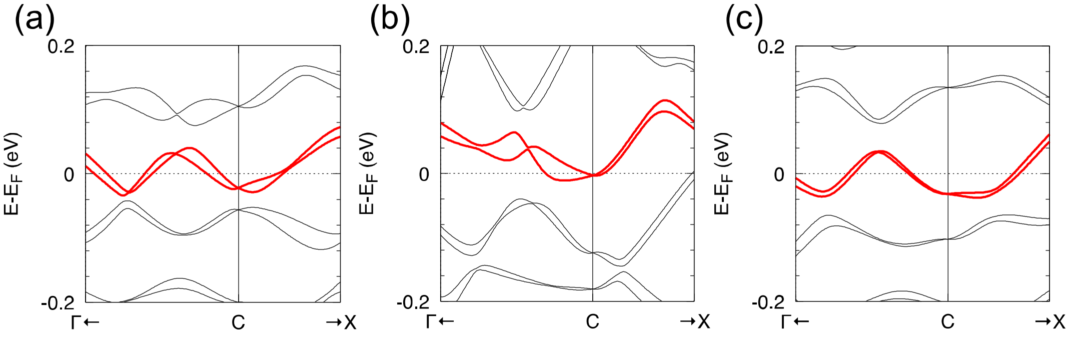

Figure S3| Band structures for NM(111)/α-Bi_2O_3. (a) NM = Cu; (b) NM=Ag; (c) NM=Au. The enlarged views of the band structures around C-point are shown through each path from C-point to the point dividing CΓ or CX line internally in the ratio 1:4.

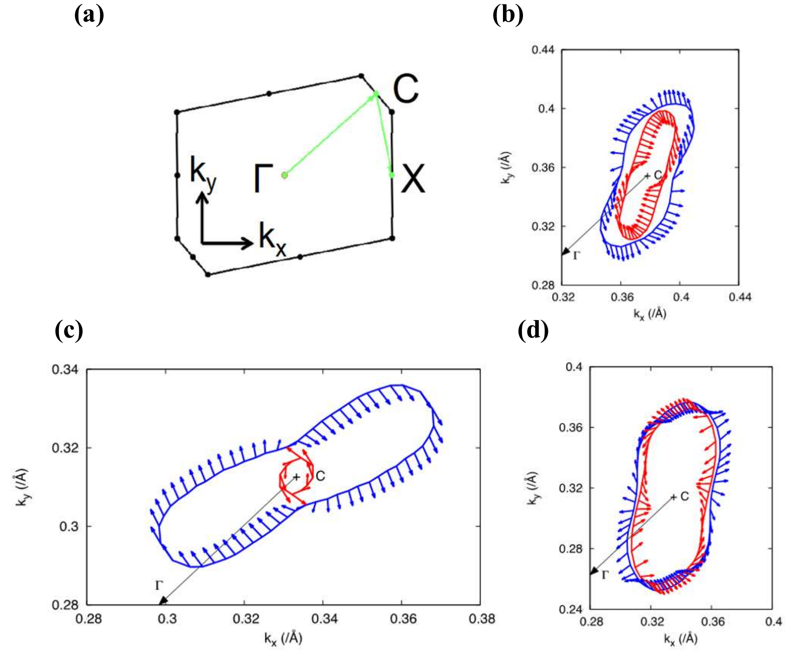

Figure S4| Atomic structure and spin textures of NM(111)/α-Bi_2O_3

(a) Schematic of the first Brillouin zone with high symmetry points. Spin textures of (b) Cu(111)/α-Bi_2O_3; (c) Ag(111)/α-Bi_2O_3; (d) Au(111)/α-Bi_2O_3. The black arrow shows CΓ line in the first Brillouin zone.
